# Novel Measures to Assess the Effects of Partial Sleep Deprivation on Sensory, Working, and Permanent Memory

**DOI:** 10.3389/fpsyg.2017.01607

**Published:** 2017-09-28

**Authors:** Dominique Gosselin, Joseph De Koninck, Kenneth Campbell

**Affiliations:** School of Psychology, University of Ottawa, Ottawa, ON, Canada

**Keywords:** sleep deprivation, cognitive impairment, attention, auditory sensory memory, visuospatial working memory, semantic memory

## Abstract

Sleepiness has repeatedly been demonstrated to affect performance on a variety of cognitive tasks. While the effects of total sleep deprivation (TSD) have been extensively studied, acute partial sleep deprivation (PSD), a more frequent form of sleep loss, has been studied much less often. The present study examined the effects of sleep deprivation on novel tasks involving classic sensory, working, and permanent memory systems. While the tasks did implicate different memory systems, they shared a need for effortful, sustained attention to maintain successful performance. Because of the novelty of the tasks, an initial study of the effects of TSD was carried out. The effects of PSD were subsequently examined in a second study, in which subjects were permitted only 4 h of sleep. A general detrimental effect of both total and PSD on accuracy of detection was observed and to a lesser extent, a slowing of the speed of responding on the different tasks. This overall effect is best explained by the often-reported inability to sustain attention following sleep loss. Specific effects on distinct cognitive processes were also observed, and these were more apparent following total than PSD.

## Introduction

More than a century of experimental data suggests that sleep is an essential component for optimal cognitive processing and that its restriction can significantly impair performance on many cognitive tasks. In an extensive review, Waters and Bucks (2011) point out that a major contributor to inter-subject variance in a large number of cognitive studies is the fact that many of these subjects will be fatigued and sleepy, often as a result of partial sleep loss. Indeed, restriction of sleep has frequently been employed as an experimental manipulation in cognitive studies to examine the extent to which various cognitive processes are affected by fatigue.

In most of these studies, subjects are totally sleep-deprived. The effect of total sleep deprivation (TSD), the complete absence of sleep over at least 24 consecutive hours, has now been extensively studied (Koslowsky and Babkoff, 1992; Pilcher and Huffcutt, 1996; Philibert, 2005; Alhola and Polo-Kantola, 2007; Lim and Dinges, 2010; Ma et al., 2015; Wickens et al., 2015). There is general consensus that this form of sleep restriction will affect tasks that are dependent on sustained attention and vigilance to maintain performance. Outside of the laboratory, individuals are however rarely totally sleep-deprived. On the other hand, acute partial sleep deprivation (PSD) is a frequent occurrence. PSD is commonly operationalized by a sleep curtailment involving less than 7 h of sleep per night (Dinges et al., 2005), and can either be experienced in an acute (one single night of insufficient sleep) or chronic (several consecutive nights of insufficient sleep) manner. In most cases, the sleep loss is caused by a delay of sleep onset time, i.e., going to bed later than the normal sleep time. Unfortunately, in spite of the pervasiveness of PSD, its acute effects on cognitive performance have not often been studied.

An early meta-analysis (Pilcher and Huffcutt, 1996) of a small number of PSD studies indicated partial sleep loss had a relatively large detrimental effect on performance. More recent studies have noted that the effects are much smaller, or absent. A possible reason for this is that the studies have employed very different cognitive tasks with methodologies that vary widely, making generalization difficult (Alhola and Polo-Kantola, 2007). A task that is however commonly used is the Psychomotor Vigilance Test (PVT; Dinges and Powell, 1985), a brief 10 min task that requires subjects to detect an infrequently and randomly presented visual stimulus. Accuracy of detection and speed of responding (reaction time, RT) typically deteriorate on this task following PSD, although the effect is smaller than for TSD (Lim and Dinges, 2008; Basner and Dinges, 2011). Unfortunately, much less is known about the effects of PSD on more complex cognitive processing. Tasks used in TSD studies have often been employed but as mentioned, methodologies vary widely. Many other studies have employed standard neuropsychological tests as a measure of cognitive functioning. There are, of course, methodological limitations to the use of standardized tests. As Killgore (2010) has pointed out, these tests were originally developed for the assessment of individuals having brain injuries and dysfunctions. Thus, although these instruments are relevant in a clinical context, the effects of sleep loss on cognitive processing may be too subtle to be detected by such standardized tests. Van Dongen et al. (2011) also note another limitation of many studies – the specific cognitive processes involved in the various tasks need to be identified to better understand the causal factors underlying the effects of sleep loss on performance. The need for a specification of cognitive functions may be especially important in the case of PSD in which the effects would be expected to be smaller and more subtle than in the case of TSD (Van Dongen et al., 2003). Many sleep deprivation studies also use pre–post designs, testing subjects prior to and following sleep deprivation. Some cognitive tasks are nevertheless subject to practice effects, performance thus improving on repeated administration of the same task (Alhola and Polo-Kantola, 2007). Although this can be mitigated by the use of a counterbalanced pre-post normal sleep condition, practice effects will nevertheless add noise to the data.

The present study examines the effects of sleep loss on tasks that involve three well-documented types of memories, sensory, short-term/working, and long-term/permanent as outlined in the Atkinson and Shiffrin (1968) model. Although each task implicated different types of memory, they all required executive support to allow attention to be focused and maintained on the task-at-hand and to inhibit the processing of distractors. This is not incidental. Lim and Dinges (2010) point out that vigilance and sustained attention are fundamental to many higher aspects of cognition. Similarly, Balkin et al. (2008) have noted that because TSD appears to affect so many different cognitive tasks, it is entirely possible that this may well be a result of a more general, non-specific inability to sustain attention. Whether PSD affects specific cognitive processes or whether its effects, like those of TSD, can be explained by a more general inability to sustain attention remains unknown. Thus, in the present study, while success on the different tasks will indeed require a general maintenance of attention and vigilance, each of the memory tasks also involves unique and specific cognitive processes.

Another goal of the present study was to develop relatively short duration tasks that could be used in clinical and applied settings. Some of the tasks employed have not, however, been used previously in sleep deprivation studies. For this reason, an initial study of the effects of TSD was run, using a relatively small sample size. Effects would thus need to be quite large and consistent to be statistically significant. Presumably, PSD would not be expected to adversely affect performance unless TSD had a large effect. A second study then examined the effects of PSD and a larger sample size was run because a smaller effect was expected.

Sensory memory was studied using a task initially developed by Schröger and Wolff (1998). Its parameters test the elaborate Näätänen (1990; Näätänen et al., 2010) model of the detection and experience of auditory change. This model assumes that the basic features of auditory sensory input are initially extracted and stored in a brief-lasting and limited sensory memory. This information is stored, maintained and retrieved automatically, and thus reflects a pre-attentive process.

In the Schröger and Wolff task, subjects are asked to discriminate the duration of equally probable short and long duration frequently occurring “standard” auditory tones. To do so requires executive support for attention to be sustained for the entire duration of the task. At rare (or “odd”) times, a feature of the standard is changed. This deviant feature is however irrelevant to the duration detection task, subjects still needing to determine whether the duration of the deviant is short or long. In the Näätänen model, a comparison is made between the extracted features of the incoming stimulus to those of the standard already stored in sensory memory. When the deviant is presented, the features fail to match those of the stored representation of the standard and stimulus change is detected. Upon detection of change, an interrupt is sent to the central executive, its amplitude varying in proportion to the extent of change. If the amplitude is sufficiently high, processing of the primary task is interrupted, and attention is switched to the processing of the potentially more relevant deviant stimulus, allowing its contents to be available to consciousness. This process thus provides a means of becoming conscious of otherwise unattended acoustic input that may be critical for survival. Objective behavioral evidence of the switch of attention away from the processing of the relevant feature of the stimulus, its duration, and toward the processing of the irrelevant feature, its pitch, is provided by performance on the task: accuracy of detection of the duration of the deviant deteriorates and speed of responding slows after the deviant is presented. Following the switch of attention, attention must be directed back to the relevant task-at-hand. This is marked by a subsequent improvement in performance.

A limited number of studies have examined the effects of sleep deprivation on sensory memory as indexed by electrophysiological measures. Electrophysiological measures have been favored because the operations of sensory memory are said to be pre-attentive and as such, subjects may not be conscious of stimulus change. Event-related potentials (ERP) provide a means of measuring the extent of pre-attentive stimulus processing. The detection of change and the operations of sensory memory are associated with a Mismatch Negativity (MMN), a negative-going ERP peaking at about 100–200 ms. Importantly, the MMN does not appear to change over repeated tested sessions and is thus not affected by practice effects (Tervaniemi et al., 1999). Zerouali et al. (2010) studied how PSD affected the MMN elicited by either a change in the frequency or the duration of the standard. In their experiment, subjects were instructed to ignore the standard and deviant auditory stimuli while engaged in a visual task. An overall ANOVA did not reveal significant differences between the MMN recorded after normal sleep and 4 h of sleep deprivation. Nevertheless, paired comparisons did indicate a significant attenuation of the MMN when subjects were permitted to sleep in the first half of the night. Zerouali et al. (2010) did not, however, examine the effects of the sleep deprivation on performance. Other studies have observed a later positivity, the P3a, often associated with the subsequent switching of attention from the task-at-hand and toward the auditory channel (Polich and Criado, 2006). Gosselin et al. (2006) tested patients with obstructive sleep apnea syndrome (OSAS) using a variation of the Schröger and Wolff paradigm. The authors suggested that long-term sleep apnea might affect the ability to detect highly relevant, but unattended stimulus change. As in the present study, subjects were asked to actively attend to a sequence of equally probable short and long duration auditory tones and to discriminate the duration of the stimuli. At rare times, a large change to the pitch of these stimuli was made, although this deviant feature was irrelevant to the duration detection task. RT was longer following presentation of the deviant compared to that of the standard stimuli for controls, but also for patients. Presumably, attention was switched from the processing of the relevant feature, the duration of the stimulus, to the processing of an irrelevant feature, its pitch. There was thus little evidence that OSAS affected the ability to switch attention to potentially highly relevant, but unattended input.

The use of the duration detection task has rarely been employed in PSD studies. While the overall active discrimination of stimulus duration is expected to decrease following sleep deprivation (because performance is dependent on sustained attention), the effects of the deviant stimulus are more difficult to predict. In the normal sleep condition, performance would also be expected to deteriorate following presentation of the deviant. Change detection is thought to occur automatically, without the need for effortful, sustained attention. Indeed, there is now considerable physiological evidence that this process occurs relatively independent of attention (Muller-Gass et al., 2006; Sussman, 2007). As such, sleep loss may not affect detection of change and thus, the presentation of the deviant should still have a detrimental effect on performance, similar to that observed after normal sleep. On the other hand, Zerouali et al. (2010) did observe an effect of PSD on the detection of auditory change. In this case, the presentation of the auditory deviant may have a reduced detrimental effect on performance.

Short-term (working) memory was examined using a modified Sternberg task. In the now classic Baddeley and Hitch (1974) model of working memory (see Baddeley, 2007 for revisions), two short-term stores were proposed, a speech-based phonological loop and a visual and spatial-based visuospatial sketchpad. In order to prevent rapid decay of speech-based information within a few seconds, articulatory rehearsal can be used to refresh and maintain the memory. In addition, visual information (written words, letters and images) can also be maintained by articulatory rehearsal by translating it into a verbal code, assuming it has a verbal label. Visuospatial working memory is, however, more complex and much less understood. The visual (“what”) and spatial (“where”) aspects may, for example, exist as separate subsystems (Logie, 1995). The mechanism by which the memory is maintained remains disputed, perhaps relying on visualization or active refreshing (Logie, 1995; Cowan, 2011; Hollingworth and Maxcey-Richard, 2013; Hu et al., 2016).

Critically, the maintenance of speech-based working memory through the articulatory rehearsal process also requires active vigilance and effort. Executive support may enable modulation of working memory capacity and maintain information in an active and quickly retrievable state (Engle, 2002). Because of the need for sustained attention, the articulatory rehearsal process should be susceptible to the effects of sleep deprivation. Most sleep deprivation studies of working memory employ visual stimuli. The results from these studies are not always consistent. While some authors report that sleep deprivation causes a decrement in performance as indicated by a decrease in accuracy and/or slower RT (Van Dongen et al., 2003; Lo et al., 2012, 2016; del Angel et al., 2015), others have failed to find an effect (Carskadon et al., 1981; Randazzo et al., 1998; Casement et al., 2006).

The nature of the tasks might explain part of these inconsistencies. In many studies, the visual stimuli (numbers, letters, geometric shapes) could be converted into a verbal code and thus maintained in working memory through the use of articulatory rehearsal. However, verbal abilities typically require executive support and can also be affected by sleep deprivation (Horne, 1988; Harrison and Horne, 1997, 1998; Harrison et al., 2000; Jiang et al., 2011; Lo et al., 2012). What might be affected by sleep deprivation is the ability to sustain the attention necessary for the functioning of articulatory rehearsal or alternately, to translate the visual stimuli into a verbal code for use with articulatory rehearsal. Forest and Godbout (2000), for example, prevented the use of articulatory rehearsal and observed that sleep deprivation led to a larger deterioration in performance on a working memory task compared to when it could be used. Other studies of total and PSD have employed Visuospatial Working Memory tasks in which a single briefly presented visual stimulus consisting of an array of colored shapes was followed immediately by a probe (e.g., Chee and Chuah, 2007; Drummond et al., 2012). As such, time restriction would not allow the amount of information contained in the visual array to be converted into a verbal code. Nevertheless, because the target occurred soon after the visual array, the memory for the array might have been retained automatically, possibly through priming or sensory memory, without the need for effortful rehearsal (Hu et al., 2016).

To overcome these problems, the present study will employ a sequence of four visual images followed by a probe. Sequential presentation has the additional advantage of yielding serial position data that are unavailable when simultaneous presentation used in other sleep deprivation studies is employed. While the most recently presented item might be retained automatically, retention of the earlier presented items should require effortful rehearsal and executive support. The visual images employed in the present study were similar to the abstract paintings used by Petrides et al. (1993). Such abstract images cannot easily be converted into a verbal code. Thus, if sleep deprivation affects working memory because of the inability to sustain attention to the task, then a deterioration in performance should be observed. On the other hand, if sleep deprivation mainly affects the ability to employ verbal codes for articulatory rehearsal, then no deterioration in performance should be observed.^1^

Long-term (permanent memory) has been the subject of many cognitive studies and reviews over the past 50 years. In the present study, a word association priming task was employed. In the usual semantic priming task, an initial word (the “prime”) is presented to the subject followed by a second word (the “target”). The subject might be asked to read the target word aloud or perhaps to make a lexical decision (whether it is a valid word or not). It has now been frequently observed that target processing is enhanced when the target word (for example, *nurse*) is preceded by a prime word to which it is semantically associated (for example, *doctor*), compared to when it is preceded by a word to which it is not semantically associated (for example, *apple*). Subjects thus respond faster and more accurately to target words that have been primed (for example, *doctor-nurse*) compared to targets that have not been primed (for example, *tree-hat*). Priming tasks are useful for the study of the effects of sleep deprivation. This is because, as previously indicated, tasks that require controlled, effortful processing and sustained attention are especially prone to the effects of sleep deprivation. On the other hand, tasks that are said to be automatic, requiring effortless processing, should be less affected. Both automatic and controlled processing have long been implicated in priming tasks and remain an area of much current dispute (e.g., Neely and Kahan, 2001; Pecher et al., 2002; Hutchison et al., 2014; Heyman et al., 2016). Automatic priming theories (Collins and Loftus, 1975; Anderson, 1983) postulate that the presentation of the prime automatically results in a spreading activation of associated words. This processing thus takes place outside of conscious awareness or intention, and occurs effortlessly without the need of sustained attention. By contrast, attention-based expectancy-prediction theories postulate that subjects can consciously use the prime to generate an expectancy for a set of associated target candidates (Posner and Snyder, 1975; Neely, 1976; Becker, 1980). The cost to the use of this strategy is that is does require active and effortful processing in order to generate possible target candidates and maintain them in working memory.

Priming and Word Association tasks have not often been used in the study of the effects of sleep deprivation. Swann et al. (2006) employed a repetitive priming task in which subjects were presented with a very short duration prime (either a valid or invalid word) and after its offset, a target. The target either matched the characters of the prime (repetitive priming) or did not. The subject’s intentional task was to determine if the target was a valid word or not. Subjects either slept normally or were totally sleep-deprived. In the normal sleep condition, RTs were faster when the prime matched the characters of the target. The authors postulated that because the initial prime was masked by the onset of the target, subjects could not have consciously identified it and must therefore have employed automatic, non-conscious processing. In agreement with the notion that TSD does not affect automatic processing, performance on the repetitive priming condition did not differ from the normal sleep condition. The matching task did not, however, require permanent memory or semantic processing.

An initial study of the effects of sleep deprivation on actual word association was carried out in our lab by López Zunini et al. (2014). Their primary interest was, however, in ERP measures of processing involved in the Word Association task. They presented subjects with target words that were either strongly, weakly, or not associated with the previously presented primes. The expected priming effect was found following normal sleep, accuracy being higher and mean RTs faster to strongly associated targets. A priming effect was also found following TSD. The ERPs during this task were, however, different in the normal and totally sleep-deprived conditions. This suggested to the authors that the priming benefit was maintained in spite of the effects of TSD by a switch of processing modes – a controlled and effortful expectancy-based search of the semantic network following normal sleep, but an automatic and effortless search following sleep deprivation. A problem with this interpretation is that it is based on the finding that the priming effect was maintained in the sleep deprivation condition – mean RTs remained longer following unassociated targets. The use of a mean RT as a dependent measure can be problematic in sleep deprivation studies. Many of these studies have pointed out that on some trials, RTs can be very slow (Dorrian et al., 2004). Such long lapses will of course skew the mean RT measure. It is possible that the maintenance of the apparent priming effect might be explained by a skewing of the mean by a few very slow responses in the unprimed condition. The median RT might thus be a better measure of central tendency of speed of responding. Tavakoli et al. (2015) replicated the word association effect with partially sleep-deprived subjects. RTs were again slower to targets that were weakly and unassociated with the previous prime, compared to those that were strongly associated. Again, the use of the mean RT as a dependent measure can be problematic. The present study will follow-up those of López Zunini et al. (2014) and Tavakoli et al. (2015), employing both mean and median RTs.

## Experiment 1 – Total Sleep Deprivation

### Materials and Methods

#### Subjects

Twelve young adult university students (six males, six females) between the ages of 20 and 31 years (*M* = 24.3, *SD* = 3.7 years) volunteered to participate in this study. All were right-handed, with good self-reported health and normal or corrected-to-normal eyesight. All subjects presented a regular sleep-wake schedule (no night or shift work) that included 7–9 h of time-in-bed with a bed time of 2200-0000 and a wake time of 0600-0800. Subjects were required to complete daily sleep diaries (Morin, 1993) two weeks before the beginning of the experimental procedures. These diaries were subsequently verified prior to the subject being accepted in the study. A medical interview was used to: (1) confirm that subjects did not have any medical conditions or were taking any medications known to affect cognitive functions, and (2) ensure that none had a history of neurological disorder. Absence of severe psychiatric disorders or sleep disorders was verified using the Mini International Neuropsychiatric Interview (MINI; Sheehan et al., 1998), the Structured Interview for Sleep Disorders (SIS-D; Schramm et al., 1993), and the Pittsburgh Sleep Quality Index (PSQI; Buysse et al., 1989).

Both the total and PSD studies were conducted following the guidelines of the Canadian Tri-Council (Health, Natural, and Social Sciences) on ethical conduct involving humans. All subjects gave written informed consent prior to the beginning of the experiment and were paid an honorarium for their participation.

#### Procedure

##### Experimental sessions

The study protocol was based on a repeated measures design. Thus, all subjects participated in two experimental sessions: one following a normal night of sleep, and one following TSD. The order of the sessions was counterbalanced across subjects and at least one week separated the two sleep conditions. This interval allowed subjects to have ample time to recover from the effects of sleep deprivation. Subjects were asked to retire for sleep and to awaken at the same times for seven consecutive nights prior to both the normal sleep and sleep deprivation sessions. Compliance with this procedure was verified by a review of daily sleep diaries that subjects had to complete for a one-week period. Subjects were also asked to abstain from alcohol and caffeine in the 24 h period prior to the start of data collection. On the sleep-deprived night, a research assistant was present at all times to ensure subjects did not sleep even for brief periods of time. Subjects engaged in quiet activities such as watching videos, reading or playing computer games for the entire duration of the night. Consistent with many other studies, data collection occurred between 0900 and 1100 the next morning. Upon coming to the lab, subjects completed the Stanford Sleepiness Scale (SSS; Hoddes et al., 1973), a 7-point rating scale (1 = awake and alert, 7 = imminent sleep onset).

##### Experimental tasks

Subjects engaged in three experimental tasks during the two sleep conditions. The order of the tasks was randomized across subjects and experimental sessions. For all three tasks, stimulus presentation, response monitoring, and timing were controlled by E-prime software (Psychology Software Tools Inc., Sharpsburg, Pennsylvania) using a PC with a Windows XP operating system. Subjects sat about 0.6 m at eye height in front of an LCD monitor. The duration of each task lasted about 10 min. In each task, subjects were asked to press a mouse button as quickly and as accurately as possible to signal their response. Performance was measured by both accuracy of responding and RT. RT was measured with a precision of 1 ms.

*Sensory Memory – Change Detection task.* In this task, subjects were presented with short and long duration stimuli and asked to determine their duration. A total of 1000 moderate intensity (70 dB SPL) auditory pure tones (1000 Hz) were presented binaurally through Sony MDR-V6 headphones. The duration of half the stimuli was 190 ms and for the other half, 310 ms. The short and long stimuli were presented in random order. A stimulus occurred every 1400 ms. Subjects were asked to press one mouse button for short duration stimuli and a second button for long duration stimuli. A response had to occur within 1200 ms. This 1000 Hz “standard” stimulus was presented on 84% of trials. A small frequency change was, however, introduced on the remaining trials. A 950 Hz “deviant” was thus presented on 16% of trials. Standards and deviants occurred in random order with the restriction that a deviant be followed by at least three presentations of the standard. Performance measures (accuracy and RT) were collapsed across short and long duration trials because accuracy of detection of a specific stimulus duration was not a concern in this study. Importantly, accuracy and RT were, however, measured separately for standards and deviant trials. In addition, recovery from the distracting effects of the presentation of the deviant were determined by observing performance on the first and second subsequent presentations of the standard stimuli following the occurrence of the deviant. The features of the first standard stimulus occurring after the deviant of course match the features of the standard occurring prior to the deviant. Nevertheless, the standard occurring after the deviant also represents stimulus change from what was presented immediately before, the actual deviant. The occurrence of the second standard, however, matches the features of that standard presented immediately before. Two blocks of 500 stimuli were presented, each block lasting 10 min. The relatively long testing time was required to ensure that enough of the rare deviants were presented to provide reliable data.

**FIGURE 1:**
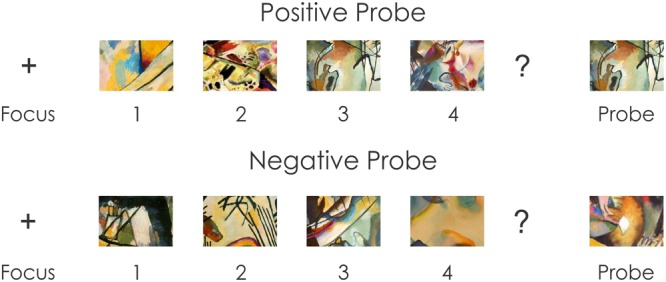
Visuospatial Working Memory task. The subject saw a fixation point for 1 s. Four different abstract images were then sequentially presented. The duration of each image was 250 ms and the time between images was 300 ms. A question mark was presented following the last item followed by a probe. The duration of the probe was also 250 ms, which occurred among the previously presented items on half the trials (positive probe) but did not occur on the other half (negative probe). The images were clips made from W, Kandinsky abstract paintings downloaded from http://www.wassilykandinsky.net/.

*Working Memory – Visuospatial Working Memory task*. In this task, subjects were presented with four different images followed by a probe. The subject’s task was to determine whether the probe was a member of the set that had just been presented. The procedure is illustrated in **Figure 1**. Each trial began with a 1000 ms duration fixation point (“+”) presented in the center of the monitor. The memory set was then presented. This memory set included four abstract colored images (clips from Kandinsky abstract paintings) that were sequentially presented. Each image lasted 250 ms. The relatively brief duration of the complex abstract image should have been insufficient to permit a conversion into a verbal code. The time between images was 300 ms, during which the subject was presented with a blank screen. After the final image of the set, a question mark appeared for 250 ms. This served to warn the subject that the probe was about to be presented. The duration of the probe was also 250 ms. Subjects were asked to push one mouse button if they decided the probe had been a member of the set and a second mouse button if they decided it had not been previously presented. Subjects had a maximum of 1000 ms to provide a response. The positive probe decisions were sorted according to the position (first to fourth) of the item within the memory set.

A total of 104 trials were presented. Different abstract images were used in each trial to obviate any practice effects. Half of the probe images were part of the memory set (positive probe), while the remaining half were not (negative probe). An equal number of positive probes were presented in either the first, second, third or fourth position of the memory set. The memory set and probe images subtended a horizontal visual angle of 15° and a vertical angle of 10°.

*Permanent Memory – Word Association task*. In this task, subjects saw an initial word (the “prime”) followed by a second word (the “target”). The subject’s task was to determine if the target word was semantically associated to the prime word. A trial began with a 2000 ms duration fixation point (“+”) presented in the center of the monitor. Next, the “prime” was presented for 200 ms. The target was presented 500 ms after the offset of the prime. This delay was sufficiently long to allow subjects to generate possible target candidates, thus permitting use of an expectancy-based cognitive strategy. The duration of the target word lasted until the subject made a response or for a maximum of 1200 ms. The next trial then began with the presentation of the fixation point. A total of 210 trials were presented.

Three different target types were presented: strongly associated (e.g., *bee-hive*), weakly associated (e.g., *cat-paw*) and unassociated (e.g., *apple-road*). The subject was asked to press one mouse button if the target was associated with the prime, and a different button if the target was not associated with the prime. Thus, the same response button was to be used for the strongly and weakly associated targets. All words were presented in lower case black font against a white background. They subtended a horizontal visual angle between 3 and 12° and a vertical angle of 1°. The Edinburgh Associative Thesaurus (EAT; Kiss et al., 1973) was used to establish the strength of the prime-target association. The extent of association for strongly associated prime-target pairs exceeded 0.60 (*M* = 0.68, *SD* = 0.10) and did not exceed 0.10 (*M* = 0.09, *SD* = 0.01) for weakly associated prime-target pairs. There was no association for the unassociated prime and target words. All prime words did, however, have a strong possible association with another target word, although only one-third of these primes were actually followed by a strongly associated target. Thus, the presentation of the prime could not be used to predict the strength of the association of the subsequent target.

A total of 420 prime-target pairs were constructed and were randomly divided into two lists that included 70 pairs of each of the prime-target association types. The strength of association between the prime and the target did not significantly differ between the two lists. Subjects were presented with both lists, a different list being presented in each experimental session. None of the words in either of the lists was repeated. Subjects did not therefore see the same prime-target pairs more than once. This procedure was established in order to minimize any learning effect. Half the subjects were presented with one list initially and the other half, with the second list.

### Data Analyses

Each of the three different tasks provided two measures of performance: accuracy of responding and speed of responding (RT). These were analyzed separately. The amount of sleep (normal, TSD) was entered as a repeated measure (within factor) for each of the three tasks.

RT was computed only on correct trials. There were too few errors to reliably analyze the error data. The subject’s mean RT was computed in each of the tasks in the normal sleep and TSD conditions. A problem with the use of the mean RT is that it can be skewed by extremely fast or, especially in the case of sleep deprivation, slow RTs (Dorrian et al., 2004; Griffith and Mahadevan, 2006). Thus, an increase in the mean RT following sleep deprivation might not reflect an overall slowing of the speed of responding, but might rather reflect a tendency toward very slow responding on only a limited number of trials. For this reason, the subject’s median RT was also computed. Some studies have indicated that sleep deprivation also increases variability of single RTs (see Dorrian and Dinges, 2006). For this reason, the standard deviation (SD) of individual subject’s RT distribution was also used as a dependent measure.

All data were inspected to detect both any missing data (failures to respond) and outliers defined by the modified *z*-score method (Iglewicz and Hoaglin, 1993). In general, the various tasks were analyzed using two-way ANOVAs with repeated measures on sleep (normal, sleep deprivation) and task conditions/stimuli. Results were considered to be significant at α = 0.05. The specific procedures will be described with each task. Previous studies have shown that sleep deprivation may result in either a deterioration or no change in performance. For this reason, a one-tailed test of significance was applied to the main effect of sleep. Assumptions of normality and homogeneity of variances were verified before conducting each ANOVA. Normality of distribution was tested with an analysis of skewness and kurtosis, as well as the Shapiro–Wilk test. Findings revealed that the underlying distribution of the sample was normal. Homogeneity of variances was assessed by the Mauchly’s test. Because a repeated measures design was used, an additional assumption, that of sphericity, was tested. When appropriate, degrees of freedom were adjusted using the Greenhouse–Geisser or Huynh–Feldt corrections, depending on the Greenhouse–Geisser estimate of sphericity value.

## Results

### Stanford Sleepiness Scale

As expected, subjective ratings of sleepiness significantly increased following TSD compared to the normal sleep condition. The mean subjective rating was 2.00 (*SD* = 0.74) following normal sleep, and 6.08 (*SD* = 0.90) following TSD, *t*(11) = 12.15, *p* < 0.001.

### Cognitive Tasks

#### Sensory Memory – Change Detection task

For this task, data are based on 11 of the 12 subjects. Performance data of one subject during TSD approximated chance level as evidenced by extreme modified *z*-score values. Data from this subject were therefore excluded from the analysis. The data were analyzed using a two-way ANOVA with repeated measures on amount of sleep (normal, TSD), and stimulus type (standard, deviant).

*Effect of presentation of the deviant*. **Table 1A** presents the effects of TSD on accuracy and speed of responding of detection of the duration of the standard and deviant stimuli.

**Table 1A:** Change Detection task: mean accuracy and RT (SD in parentheses) as a function of the type of stimulus (standard or deviant), and the amount of sleep.

Type of auditory stimulus	Accuracy (%)		RT (ms)	
	Sleep condition		Sleep condition	
	NS	TSD	NS	TSD
Standard before deviant	0.89 (0.05)	0.75 (0.08)	575 (53)	579 (51)
Deviant	0.78 (0.08)	0.70 (0.12)	615 (63)	599 (53)

*NS, normal sleep; TSD, total sleep deprivation.*

#### Accuracy

Overall accuracy of detection (of both the standard and deviant) significantly decreased following TSD, *F*(1,10) = 19.18, *p* = 0.001; $\eta_{p}^{2}$= 0.66. An overall main effect of stimulus type was also found, *F*(1,10) = 8.68, *p* = 0.015; $\eta_{p}^{2}$= 0.47. As expected, accuracy of discrimination of the duration of the stimulus significantly decreased following presentation of the deviant. Importantly, a significant interaction between sleep and the type of auditory stimulus was also observed, *F*(1,10) = 5.68, *p* = 0.04; $\eta_{p}^{2}$= 0.36. In the normal sleep condition, accuracy of detection of stimulus duration significantly decreased by 11.2% following presentation of the deviant. In the TSD condition, presentation of the deviant resulted in only a 5.2% deterioration in performance and in this case, the standard-deviant difference was not significant.

The number of failures to respond also significantly increased following TSD, *F*(1,10) = 6.75, *p* = 0.03; $\eta_{p}^{2}$= 0.40. The main effect of type of auditory stimulus was not found to be significant, *F* < 1, nor was the interaction between sleep and type of auditory stimulus, *F* < 1.

#### Speed of responding

No main effect of sleep was observed for the mean and median RTs, *F* < 1 in both cases, and for the SD of the individual mean RT, *F*(1,10) = 4.57, *p* = 0.06. As expected, mean RT did significantly increase following presentation of the deviant, *F*(1,10) = 21.71, *p* = 0.001; $\eta_{p}^{2}$= 0.69. However, a significant interaction between sleep and type of stimulus was observed for both mean and median RTs, *F*(1,10) = 10.12, *p* = 0.01; $\eta_{p}^{2}$= 0.50, and *F*(1,10) = 7.47, *p* = 0.02; $\eta_{p}^{2}$= 0.43, respectively. Again, a detrimental effect of presentation of the deviant was apparent following normal sleep but not following TSD. Thus, RTs significantly slowed after presentation of the deviant following normal sleep, but were not significantly slower following TSD. The interaction between sleep and type of stimulus was not significant for the SD of the individual mean RT, *F* < 1.

*Recovery following presentation of the deviant*. Recovery from the deterioration effect of the deviant was quantified by examining performance on the two standards occurring after the presentation of the deviant. **Table 1B** presents findings related to recovery following presentation of the deviant and the effects of the amount of sleep.

**Table 1B:** Change Detection task: mean accuracy and RT (SD in parentheses) as a function of recovery from presentation of the deviant, and the amount of sleep.

Type of auditory stimulus	Accuracy (%)		RT (ms)	
	Sleep condition		Sleep condition	
	NS	TSD	NS	TSD
Deviant	0.78 (0.08)	0.70 (0.12)	615 (63)	599 (53)
Standard after deviant 1	0.82 (0.11)	0.72 (0.15)	602 (65)	597 (69)
Standard after deviant 2	0.87 (0.09)	0.78 (0.12)	601 (72)	594 (59)

*NS, normal sleep; TSD, total sleep deprivation.*

#### Accuracy

The previous analysis indicated that there was evidence of an interactive effect of the deviant and the amount of sleep on accuracy of detection. Accuracy did improve (or “recover”) following the subsequent presentation of the standards, and showed an overall improvement of about 8.5% (regardless of the amount of sleep) when the second subsequent standard was presented. This main effect of stimulus type did not, however, attain significance, *F*(2,20) = 1.78, *p* = 0.19. A significant overall main effect of sleep remained, *F*(1,10) = 9.67, *p* = 0.01; $\eta_{p}^{2}$= 0.49. TSD was associated with a significant 8.9% decrease in the overall accuracy of detection of both the deviant and subsequent standards. The interaction between sleep and type of auditory stimulus was, however, not significant, *F* < 1.

The number of failures to respond did not significantly differ between the standard and deviant after either normal sleep or TSD. A subsequent analysis of recovery from the effects of the deviant was therefore not warranted.

#### Speed of responding

The previous analysis indicated an interactive effect of the deviant and the amount of sleep on the speed of responding. The deviant resulted in a delayed RT following normal sleep but had only a small effect following TSD. A comparison of RTs to the standards following presentation of the deviant did not reveal significant recovery for the mean or median RT: neither varied as a function of sleep or as a function of stimulus type, *F* < 1 in both cases.

#### Working Memory – Visuospatial Working Memory task

For this task, data are based on 11 of the 12 subjects. Performance data of one subject was not available due to technical difficulties. For the positive probes, the data were analyzed using a two-way ANOVA with repeated measures on amount of sleep (normal, TSD), and serial position (1st…4th). For the negative probes, a *t*-test with repeated measures on amount of sleep was applied. **Table 2** presents the effects of TSD on accuracy and speed of responding during the Visuospatial Working Memory task.

**Table 2:** Visuospatial Working Memory task: mean accuracy and RT (SD in parentheses) for both positive and negative probes as a function of the amount of sleep.

In memory set?	Position	Accuracy (%)		RT (ms)	
		Sleep condition		Sleep condition	
		NS	TSD	NS	TSD
Yes	1	0.50 (0.11)	0.49 (0.16)	697 (105)	647 (88)
	2	0.49 (0.18)	0.38 (0.19)	678 (101)	638 (124)
	3	0.64 (0.19)	0.60 (0.14)	645 (109)	628 (120)
	4	0.90 (0.08)	0.80 (0.09)	548 (77)	545 (94)
No		0.79 (0.08)	0.69 (0.12)	643 (89)	646 (81)

*NS, normal sleep; TSD, total sleep deprivation.*

*Accuracy*. For positive probes (i.e., probes that were among the memory set), a significant main effect of serial position on accuracy of detection (i.e., regardless of the amount of sleep) was observed, *F*(3,30) = 26.13, *p <*0.001; $\eta_{p}^{2}$= 0.72. A recency effect was apparent such that, in general, accuracy gradually improved when the item to-be-remembered was presented temporally closer to the actual positive probe. Importantly, a main effect of sleep was also found for positive probes, *F*(1,10) = 14.36, *p* = 0.002; $\eta_{p}^{2}$= 0.59. Accuracy decreased by an average of 6.5% following TSD. The interaction between serial position and sleep did not reach statistical significance for positive probes, *F*(3,30) = 1.73, *p* = 0.09. A significant effect of sleep was also found for negative probes (i.e., probes that were not among the memory set), *t*(10) = 2.72, *p* = 0.01; *d* = 0.82. Accuracy decreased by 9.9% in the TSD condition.

A main effect of serial position was again observed for failures to respond to positive probes, *F*(3,30) = 5.17, *p* = 0.003; $\eta_{p}^{2}$= 0.34. The number of failures to respond to the positive probes increased by 10.5% following TSD. Similarly, for negative probes, a 4.3% increase in the number of failures to respond was observed following TSD. Both increases were found to be significant, *F*(1,10) = 12.05, *p* = 0.003; $\eta_{p}^{2}$= 0.55, and *t*(10) = 2.77, *p* = 0.01; *d* = 0.84, respectively. No interaction between serial position and sleep was found for positive probes, *F <* 1.

*Speed of responding*. For positive probes, a significant main effect of serial position on mean RT was found, *F*(3,30) = 20.88, *p <* 0.001; $\eta_{p}^{2}$= 0.68. A recency effect was again apparent such that RTs became faster when the item to-be-remembered was presented temporally closer to the actual positive probe. A main effect of sleep was not found to be significant for positive probes, either for the mean or median RT, *F* < 1 in both cases, or the SD of the individual mean RT, *F*(1,10) = 2.09, *p* = 0.09. Similarly, the interaction between serial position and sleep was not significant, *F* < 1 for the mean and median RTs, and *F*(3,30) = 1.82, *p* = 0.08 for the SD of the individual mean RT. The effect of sleep was again not significant for the speed of responding to negative probes, *t* < 1 for the mean and median RTs, and *t*(11) = 1.35, *p* = 0.10 for the SD of the individual mean RT.

#### Permanent Memory – Word Association task

The data for this task were also analyzed using a two-way ANOVA with repeated measures on the amount of sleep (normal, TSD), and strength of word association (strong, weak, unassociated). **Table 3** presents the effects of TSD on accuracy and speed of responding during the Word Association task.

**Table 3:** Word Association task: mean accuracy and RT (SD in parentheses) as a function of the strength of association between the prime and target, and the amount of sleep.

Prime-target association	Accuracy (%)		RT (ms)	
	Sleep condition		Sleep condition	
	NS	TSD	NS	TSD
Strong	0.91 (0.08)	0.77 (0.20)	608 (93)	658 (119)
Weak	0.77 (0.11)	0.69 (0.19)	670 (115)	709 (126)
Unassociated	0.84 (0.13)	0.69 (0.21)	737 (81)	782 (103)

*NS, normal sleep; TSD, total sleep deprivation.*

*Accuracy*. The expected significant main effect of the strength of word association between the prime and the subsequent target word was found, *F*(1.11,12.23) = 8.64, *p* = 0.006; $\eta_{p}^{2}$= 0.44. Accuracy was significantly higher for targets that were strongly associated with the prime, regardless of the amount of sleep. A main effect of sleep was also found, *F*(1,11) = 3.68, *p* = 0.04; $\eta_{p}^{2}$= 0.25. Accuracy decreased by an average of 12.7% following TSD. The interaction between sleep and the extent of word association did not reach statistical significance, *F*(1.27,13.96) = 1.24, *p* = 0.15.

A main effect of the strength of association was also found for failures to respond, *F*(2,22) = 11.96, *p <* 0.001; $\eta_{p}^{2}$= 0.52. The number of failures to respond was significantly reduced for strongly associated targets, in comparison to weakly associated and unassociated targets. A main effect of TSD was also apparent for the number of failures to respond, *F*(1,11) = 4.61, *p* = 0.03; $\eta_{p}^{2}$= 0.30. The number of failures to respond increased by 9.6% following TSD. The interaction between the extent of association and sleep was not significant, *F*(2,22) = 2.22, *p* = 0.07.

*Speed of responding*. A significant strength of word association main effect was also found for RT, *F*(2,22) = 54.73, *p <* 0.001; $\eta_{p}^{2}$= 0.83. RT was much faster for strongly associated than weakly and unassociated targets. RT tended to be slower following TSD. This increase in RT following TSD was, however, not significant for either the mean or median RT, or the SD of the individual mean RT, *F*(1,11) = 1.85, 2.44 and 0.97, respectively, *p* = 0.10, 0.07 and 0.17, respectively. The interaction between sleep and the extent of association was also not significant for the three measures of speed of responding, *F* < 1 in all cases.

## Experiment 2 – Partial Sleep Deprivation

### Introduction

Total sleep deprivation resulted in a significant decline in performance on all tasks regardless of the specific type of memory. This was especially the case for accuracy of performance. Because of the consistent effect of TSD, all tasks were again included in the study of PSD. It was expected that the overall effects on performance would be reduced for partial compared to TSD.

### Materials and Methods

#### Subjects

Because of the expected smaller effect of PSD, a larger sample size was employed in this study. Eighteen young adult university students (nine males, nine females) between the ages of 20 and 32 years (*M* = 23.7, *SD* = 3.3 years) volunteered to participate. All were right-handed, with good self-reported health and normal or corrected-to-normal eyesight. All subjects presented a regular sleep-wake schedule (no night or shift work) that included 7–9 h of time-in-bed with a bed time of 2200-0000 and a wake time of 0600-0800. Other rejection criteria were identical to those used in Experiment 1.

#### Procedure

##### Experimental sessions

Experimental procedures were similar to those used in Experiment 1. On the sleep-deprived night, subjects were permitted to go to bed at 0300 and were awakened at 0700. In order to ensure adherence, subjects were required to wear a wrist actigraph on this night and asked to leave voicemail call-ins every hour. Subsequent analyses of the voicemail messages and actigraph data indicated that subjects followed this sleep schedule. While awake at night, subjects engaged in quiet activities such as watching videos, reading or playing computer games for the entire duration of the night. Data collection began the following morning between 0900 and 1100. Upon coming to the lab, subjects completed the SSS (Hoddes et al., 1973).

##### Experimental tasks

Subjects participated in four experimental tasks during the two sleep conditions. The order of the tasks was randomized across subjects and experimental sessions. The tasks administered were the same as those used in the TSD experiment (Change Detection, Visuospatial Working Memory, and Word Association tasks). A fourth task, the PVT, was also administered. The PVT is considered to be the gold standard for the assessment of the effects of sleep loss on cognition (Killgore, 2010), and has been shown to be sensitive even to the effects of PSD (Lim and Dinges, 2008; Basner and Dinges, 2011). By contrast, it was not known if the other tasks would be affected by PSD.

*Psychomotor Vigilance Test*. In this task, subjects were asked to detect a visual stimulus, presented at random times. A trial began with a fixation point (“+”) presented in the center of the monitor. This was replaced by a 15 mm diameter black circle lasting 1 s. The onset of the circle varied randomly between 2 and 10 s (on average, every 6 s) after the onset of the fixation point. The subject was asked to press the left mouse button as quickly as possible upon detection of the circle. Feedback then informed the subject that a correct response had occurred and the actual RT (in ms) was displayed. If subjects failed to respond, a feedback message “no response was detected” was displayed. The feedback message also lasted 1 s after which the next trial was initiated with the presentation of the fixation point. The task lasted about 10 min. Performance was again measured in terms of accuracy of responding and RT. The mean and median RTs, and variability in individual subject’s RTs were again employed. In addition, researchers often examine “lapses” in performance (Lim and Dinges, 2008). The number of lapses, defined as RTs greater than 500 ms, was thus also computed.

#### Data Analyses

Statistical procedures employed for the Change Detection, the Visuospatial Working Memory, and the Word Association tasks were the same as those used in the initial experiment. The PVT was analyzed using a within measures *t*-test with repeated measures on sleep (normal, PSD). One-tailed tests of significance were applied because many previous studies have reported a deterioration in performance following sleep deprivation.

### Results

#### Stanford Sleepiness Scale

Subjective ratings of sleepiness significantly increased following PSD compared to the normal sleep condition. The mean subjective rating was 1.89 (*SD* = 0.68) following normal sleep and 3.33 (*SD* = 0.97) following PSD, *t*(17) = 5.88, *p* < 0.001.

#### Cognitive Tasks

##### Sensory Memory – Change Detection task

For this task, data are based on 17 of the 18 subjects. Performance of one subject was at chance level following PSD, as revealed by extreme modified *z*-score values. Data from this subject were therefore excluded from the analysis.

*Effect of presentation of the deviant*. **Table 4A** presents the effects of PSD on accuracy and speed of responding of detection of the duration of the standard and deviant stimuli.

**Table 4A:** Change Detection task: mean accuracy and RT (SD in parentheses) as a function of the type of stimulus (standard or deviant), and the amount of sleep.

Type of auditory stimulus	Accuracy (%)		RT (ms)	
	Sleep condition		Sleep condition	
	NS	PSD	NS	PSD
Standard before deviant	0.84 (0.09)	0.77 (0.14)	526 (50)	501 (71)
Deviant	0.69 (0.10)	0.66 (0.14)	628 (55)	634 (60)

*NS, normal sleep; PSD, partial sleep deprivation.*

##### Accuracy

The usual overall detrimental effect of the deviant stimulus was replicated in the PSD study. Thus, a main effect of stimulus type was observed, *F*(1,16) = 24.57, *p <* 0.001; $\eta_{p}^{2}$= 0.61, with accuracy decreasing after presentation of the deviant, regardless of the amount of sleep. This effect was greater following normal sleep than following PSD, but the interaction was not significant, *F*(1,16) = 2.35, *p* = 0.15. Rather, a significant main effect of sleep was observed, *F*(1,16) = 4.48, *p* = 0.05; $\eta_{p}^{2}$= 0.22, such that accuracy of detection of stimulus duration decreased by an average of 5.3% following PSD regardless of whether a standard or deviant was presented.

A main effect of sleep was again found for the failures to respond, *F*(1,16) = 7.18, *p* = 0.02; $\eta_{p}^{2}$= 0.31. These occurred 5.4% more often in the PSD than in the normal sleep condition. The main effect of type of auditory stimulus was also found to be significant, *F*(1,16) = 23.40, *p <* 0.001; $\eta_{p}^{2}$= 0.59. The number of failures to respond increased by 3.0% following presentation of the deviant. The interaction between sleep and type of auditory stimulus was not significant, *F* < 1.

##### Speed of responding

The interaction between the amount of sleep and the type of auditory stimulus was not significant, either for the mean *F*(1,16) = 3.60, *p* = 0.08, the median *F*(1,16) = 1.98, *p* = 0.18, or the SD of single responses *F*(1,16) = 1.11 *p* = 0.31. The difference in overall RT (the combined standard and the deviant RT) did not significantly vary as a result of the amount of sleep for either the mean or the median RT, *F* < 1 in both cases. The SD of the individual mean RT was, however, significantly larger following PSD, *F*(1,16) = 12.66, *p* = 0.003; $\eta_{p}^{2}$= 0.44. There was more variance in the PSD condition. The main effect of type of auditory stimulus was again replicated for the mean *F*(1,16) = 75.45, *p <* 0.001; $\eta_{p}^{2}$= 0.83, the median *F*(1,16) = 29.22, *p <* 0.001; $\eta_{p}^{2}$= 0.65, and the SD of the individual mean RT *F*(1,16) = 73.51, *p <* 0.001; $\eta_{p}^{2}$= 0.82.

*Recovery following presentation of the deviant*. A significant main effect of the type of stimulus was found. Performance was poorer after presentation of the deviant, regardless of the amount of sleep. However, there was little evidence of an interaction: the deterioration in performance after occurrence of the deviant was not reduced in the PSD compared to the normal sleep condition. **Table 4B** presents findings related to recovery following presentation of the deviant and the effects of the amount of sleep.

**Table 4B:** Change Detection task: mean accuracy and RT (SD in parentheses) as a function of recovery from presentation of the deviant, and the amount of sleep.

Type of auditory stimulus	Accuracy (%)		RT (ms)	
	Sleep condition		Sleep condition	
	NS	PSD	NS	PSD
Deviant	0.69 (0.10)	0.66 (0.14)	628 (55)	634 (60)
Standard after deviant 1	0.81 (0.13)	0.79 (0.12)	601 (45)	608 (55)
Standard after deviant 2	0.83 (0.09)	0.73 (0.18)	622 (45)	627 (51)

*NS, normal sleep; PSD, partial sleep deprivation.*

##### Accuracy

A significant interaction between sleep and type of auditory stimulus was found, *F*(2,32) = 5.71, *p* = 0.008; $\eta_{p}^{2}$= 0.26. The source of this interaction was, however, complex. Immediately following presentation of the deviant, accuracy significantly improved following presentation of the initial standard in both the normal sleep and PSD conditions. However, accuracy then significantly decreased after presentation of the subsequent (second) standard, but only in the PSD condition. Accuracy continued to improve in the normal sleep condition.

A main effect of sleep was again found to be significant for the failures to respond, *F*(1,16) = 6.76, *p* = 0.02; $\eta_{p}^{2}$= 0.30. These occurred 5.5% more often in the PSD condition than in the normal sleep condition. The main effect of type of auditory stimulus was also significant for the failures to respond, *F*(2,32) = 12.51, *p <* 0.001; $\eta_{p}^{2}$= 0.44. Thus, fewer failures to respond occurred for the stimuli following the deviant. This was, however, independent of the amount of sleep. The interaction between sleep and type of auditory stimulus was not significant, *F*(2,32) = 2.76, *p* = 0.08.

##### Speed of responding

The speed of responding (RT) to both the standards and deviants was not significantly affected by PSD. A follow-up to examine the extent of recovery from the deviant was therefore not carried out.

##### Working Memory – Visuospatial Working Memory task

**Table 5** presents the effects of PSD on accuracy and speed of responding during the Visuospatial Working Memory task.

**Table 5:** Visuospatial Working Memory task: mean accuracy and RT (SD in parentheses) for both positive and negative probes as a function of the amount of sleep.

In memory set?	Position	Accuracy (%)		RT (ms)	
		Sleep condition		Sleep condition	
		NS	PSD	NS	PSD
Yes	1	0.41 (0.24)	0.42 (0.23)	712 (114)	708 (95)
	2	0.47 (0.22)	0.50 (0.22)	708 (84)	711 (73)
	3	0.65 (0.22)	0.61 (0.25)	663 (63)	652 (82)
	4	0.89 (0.09)	0.88 (0.14)	571 (67)	574 (62)
No		0.79 (0.11)	0.72 (0.17)	660 (60)	666 (54)

*NS, normal sleep; PSD, partial sleep deprivation.*

*Accuracy*. A significant main effect of serial position on accuracy was again observed for positive probes, *F*(3,51) = 62.95, *p <* 0.001; $\eta_{p}^{2}$= 0.79. A recency effect was also apparent such that accuracy gradually improved when the item to-be-remembered was presented temporally closer to the actual positive probe. Importantly, neither the main effect of sleep nor the interaction of serial position and sleep was found to be significant, *F* < 1 in both cases. Similar to what was observed in the TSD study, PSD did have a significant effect on the responses to negative probes, accuracy decreasing by 7.6%, *t*(17) = 1.73, *p* = 0.05; *d* = 0.41.

The findings with respect to failures to respond in the PSD condition were also similar to those of TSD. Thus, a main effect of serial position was again found for positives probes, *F*(3,51) = 7.11, *p <* 0.001; $\eta_{p}^{2}$= 0.30. Importantly, PSD resulted in a 4.7% increase in failures to respond for positive probes, and a 7.0% for negative probes. These increases in the number of failures to respond were significant for both positive, *F*(1,17) = 4.32, *p* = 0.03; $\eta_{p}^{2}$= 0.20, and negative probes, *t*(17) = 1.77, *p* = 0.05; *d* = 0.44. No interaction between serial position and sleep was found for positive probes, *F*(3,51) = 1.42, *p* = 0.12.

*Speed of responding*. An overall significant main effect of serial position on mean RT was again observed for positive probes, *F*(2.24,38.09) = 38.90, *p <* 0.001; $\eta_{p}^{2}$= 0.70. A recency effect was also apparent such that RT was faster when the item to-be-remembered was presented temporally closer to the actual positive probe. Similar to what was found with the TSD study, PSD also did not significantly affect the speed of responding to positive probes for the mean RT, median RT and the SD of the individual mean RT, *F* < 1 for all three measures. The interaction between serial position and sleep was again not significant, *F* < 1 for all three measures. Mean and median RTs were also not significantly affected by the amount of sleep following presentation of the negative probe, *t* < 1 in both cases. The variance around the individual mean RT was, however, significantly larger in the PSD condition, *t*(17) = 2.75, *p* = 0.007; *d* = 0.67.

#### Permanent Memory – Word Association task

**Table 6** presents the effects of PSD on accuracy and speed of responding during the Word Association task.

**Table 6:** Word Association task: mean accuracy and RT (SD in parentheses) as a function of the strength of association between the prime and target, and the amount of sleep.

Prime-target association	Accuracy (%)		RT (ms)	
	Sleep condition		Sleep condition	
	NS	PSD	NS	PSD
Strong	0.92 (0.08)	0.85 (0.14)	622 (98)	629 (104)
Weak	0.74 (0.11)	0.70 (0.16)	695 (100)	712 (102)
Unassociated	0.87 (0.11)	0.82 (0.14)	724 (102)	747 (103)

*NS, normal sleep; PSD, partial sleep deprivation.*

*Accuracy*. A main effect of the strength of association was, as in the TSD, found for accuracy of responding, *F*(2,34) = 29.83, *p <* 0.001; $\eta_{p}^{2}$= 0.64. Accuracy significantly decreased for weakly associated and unassociated targets. The main effect of sleep was not, however, significant, *F*(1,17) = 2.35, *p* = 0.07. The interaction between sleep and the strength of word association was not significant, *F* < 1.

A main effect of the strength of word association was again found to be significant for failures to respond, *F*(2,34) = 7.27, *p* = 0.001; $\eta_{p}^{2}$= 0.30. The number of failures to respond was much lower for strongly associated targets. PSD did not significantly alter the number of failures to respond, *F*(1,17) = 1.50, *p* = 0.12. The interaction between the extent of association and sleep was not significant, *F*(1.64,27.95) = 1.28, *p* = 0.14.

*Speed of responding*. The PSD findings were similar to those of TSD. The extent of word association again had the predicted significant main effect on mean RT, *F*(1.40,23.79) = 56.85, *p <* 0.001; $\eta_{p}^{2}$= 0.77. The effect of PSD on speed of responding (mean RT, median RT, and variability of RT) was not significant, *F* < 1, *F*(1,17) = 1.21, *p* = 0.14, and *F*(1,17) = 2.26, *p* = 0.08, respectively. Moreover, the interaction between sleep and the strength of association was also not significant for the mean RT, *F*(2,34) = 1.05, *p* = 0.18, the median RT *F*(2,34) = 1.71, *p* = 0.10, or the variability of RT *F* < 1.

#### Psychomotor Vigilance Test

**Table 7** presents the effects of PSD on accuracy and speed of responding during the PVT.

**Table 7:** Psychomotor Vigilance Test: mean accuracy and RT (SD in parentheses) as a function of the amount of sleep.

Accuracy (%)		RT (ms)	
Sleep condition		Sleep condition	
NS	PSD	NS	PSD
0.99 (0.01)	0.97 (0.03)	336 (48)	375 (56)

*NS, normal sleep; PSD, partial sleep deprivation.*

*Accuracy*. Overall accuracy was very high in both the normal sleep and the PSD conditions. Nevertheless, PSD had a significant detrimental effect on accuracy, *t*(17) = 3.00, *p* = 0.005; *d* = 0.73. Accuracy decreased by 2.4% in the PSD condition.

*Speed of responding*. Partial sleep deprivation was associated with a significant slowing of the speed of responding, whether measured by mean RT, *t*(17) = 4.26, *p <* 0.001; *d* = 1.03, or median RT, *t*(17) = 5.06, *p <* .001; *d* = 1.23. The SD of individual RT also significantly increased following PSD, *t*(17) = 2.34, *p* = 0.02; *d* = 0.57. A significant 6.7% increase in the number of lapses (i.e., RTs longer than 500 ms) was observed following PSD, *t*(17) = 3.30, *p* = 0.002; *d* = 0.80.

## Discussion

The primary purpose of the present study was to examine the effects of 4 h of sleep loss on cognitive function. The consequences of TSD have been studied extensively and several studies have indicated that it has a large effect on many cognitive tasks, particularly if the task requires sustain attention and vigilance for successful completion. Outside of the laboratory, few individuals actually experience TSD. By contrast, partial sleep loss is common, but its effects remain poorly understood. Many different tasks have been run in previous studies but the cognitive operations involved in these tasks are often poorly understood. For this reason, three tasks involving distinct and relatively well-understood types of memories (sensory, working, and permanent) were run. Because these specific tasks have not been employed in sleep deprivation studies, the effects of TSD were examined initially. Performance deteriorated for each task.

A follow-up study of the effects of PSD was subsequently carried out. In general, performance again deteriorated but the effects were not always statistically significant. The present study thus provides strong support for the claim that loss of sleep, even if it is only for 4 h, will result in a deterioration in performance on cognitive tasks that demand effortful, sustained attention and vigilance for their successful completion. This assertion is further supported by the results of the PVT: PSD was associated with a lower accuracy of target detection, a slower RT to these targets, and more frequent lapses in comparison to normal sleep condition scores.

### Sustained Attention and Vigilance

The different tasks used in this study did share a common need to maintain attention and vigilance for successful performance. When subjects were totally sleep-deprived, a main effect of sleep was found on accuracy of detection and failures to respond for each of the tasks. Such lapses have frequently been reported in other TSD studies (Lim and Dinges, 2008). TSD did not, however, have a significant overall main effect for either the mean or median RT in either the Visuospatial Working Memory or Word Association task. Importantly, similar trends were observed following partial sleep loss. Accuracy of performance decreased but, for certain tasks, the difference compared to normal sleep was not significant. Again, the difference in the mean and median RTs between normal sleep and PSD conditions across the various tasks was not as marked. In brief, performance as measured by accuracy of detection showed a consistent reduction on all tasks following sleep loss, but performance as measured by speed of responding did not show a consistent slowing. It is possible that the differences in the performance measures may reflect the often-observed speed-accuracy trade-off in which the cost of faster responding is a risk of more errors. Following sleep loss, in order to maintain speed of responding, accuracy may have been sacrificed. De Gennaro et al. (2001) have also noted that TSD affects accuracy more than speed of responding when the experimenter controls the timing of stimulus presentation, rather than allowing the subject to do so. More recently, however, Lim and Dinges (2010) in their elaborate meta-analysis of several studies, concluded that TSD did not bias subjects toward either faster or more accurate responding.

While a general effect on the different tasks was observed regardless of the distinct cognitive processes involved in each, there were also more specific effects. For example, processes that are thought to occur automatically and are not dependent on either the direction or strength of attention were also significantly affected by TSD and to some extent by PSD. The detection of acoustic change, thought to be made automatically, was compromised by sleep deprivation. Similarly, in the Visuospatial Working Memory task, memory for the most recently presented item was affected by TSD. The storage and retrieval of this item is much less dependent on attentional resources than earlier items. Again, the differences within tasks were more apparent following total than PSD. Each task will therefore be considered separately.

### Sensory Memory – Change Detection Task

In both the total and PSD studies, the normal sleep condition provided strong evidence that the deviant was detected and that the processing of the features of this deviant resulted in a deterioration in performance. Accuracy of detection of the duration of the deviant declined and the time to make this detection was slower relative to that of the duration of the standard. This deterioration in performance is best explained by an automatic switch of attention from the task-at-hand, i.e., the detection of the duration of the stimulus to the processing of a potentially more relevant feature, its frequency.

Total sleep deprivation did affect the detection of acoustic change. The operations of this process are thought be independent of attention. Thus, while overall deterioration in performance on the duration detection could be explained by an inability to sustain attention, the specific effects of the deviant could not. A significant interaction between sleep and type of auditory stimulus on both the accuracy and speed of detection was observed. In the normal sleep condition, as expected, accuracy of detection did significantly decline following the presentation of the deviant. It did not significantly decline in the TSD condition. Similarly, both the mean and median RTs significantly slowed following the presentation of the deviant in the normal sleep condition, but not in the TSD condition. In other words, in totally sleep-deprived subjects, a small frequency change to a rarely occurring auditory stimulus is less likely to result in a switch of attention away from the task-at-hand. This has both negative and positive implications. The purpose of the attention switch is to provide a means for observers to become aware of potentially highly relevant, but unattended stimulus input, and subsequently, to take appropriate action. The present results suggest that this ability may be compromised by TSD. This has serious consequences. A number of warning systems include auditory alarms. It may be difficult, for example, for a totally sleep-deprived automobile driver to detect the sound of a horn from another automobile or for an airline pilot to detect warning alarms. Nevertheless, most of this input turns out to be, in fact, irrelevant and thus the switch of attention results in needless distraction from the cognitive task-at-hand.

In the PSD study, a main effect of type of stimulus was again found. The deviant stimulus did result in a deterioration in performance in terms of accuracy, failures to respond, and RT, in both the normal and PSD conditions. Importantly, in spite of only having 4 h of sleep, performance significantly deteriorated compared to normal sleep. In the TSD study, the deterioration in performance following the presentation of the deviant was less marked in the sleep deprivation compared to normal sleep condition. The trend was similar in the PSD study for accuracy of detection, the deviant having a reduced effect following PSD compared to normal sleep. The interaction between sleep and stimulus was, however, not significant.

There was little evidence of recovery from the detrimental effects of the deviant in the TSD condition. On the other hand, accuracy of detection of the initial standard following presentation of the deviant did improve in both the normal sleep and PSD conditions. This improvement was, however, sustained for the subsequent standard only in the normal sleep condition. Recovery from the detrimental effects of the deviant does nevertheless appear to take longer following total than PSD. This might, however, be related to the fact that the deviant had a much smaller detrimental effect in the PSD condition, and thus the need for a “recovery” of performance was reduced.

### Working Memory – Visuospatial Working Memory Task

In the TSD and PSD studies, subjects were presented with a sequence of four abstract images that presumably would have been difficult to encode verbally. How memory for these images is maintained remains a subject of debate but probably could not involve articulatory rehearsal. Nevertheless, to do so would presumably require executive support to sustain attention. This notion is supported by the present findings. Even though articulatory rehearsal was not possible, a main effect of TSD on accuracy of detection and failure to respond to both positive and negative probes was still observed. A strong recency effect was also found. In the normal sleep conditions, this observation is not surprising and is consistent with many other studies. It was also apparent, however, in both the total and PSD conditions. The fact that the recency effect is maintained after even TSD is consistent with the claim that memory for the last item in a sequence may be automatically accessible without the need for rehearsal or executive support (Allen et al., 2014). Still, performance for the recent item did deteriorate following TSD compared to normal sleep. Hu et al. (2016) note that while the most recent item in a sequence may be automatically accessible, it can still benefit from attention and executive support. Chee and Chuah (2007) also point out that sleep deprivation may be associated with degraded perceptual processing and this perceptual processing may also benefit from attention. As such, in the present study, the poorer performance for the last item in the sequence may be related to a more basic impaired perception of this image rather than an effect on memory. The locus of the effect of TSD on the visuospatial aspect of working memory will require further investigation.

The effect of PSD was more subtle. Memory for the positive probes was not significantly affected. Detection of negative probes was nevertheless significantly poorer. There was also evidence of greater uncertainty in the PSD compared to the normal sleep condition. Subjects failed to respond to both positive and negative probes more often. In cases of uncertainty when subjects did respond, their response might have reflected a bias toward guessing that a probe had been previously presented, resulting in a high hit rate (correctly detecting positive probes) but at a cost of a high false positive rate (failing to correctly reject negative probes). It is difficult to explain why subjects might favor one type of decision over another. Unfortunately, previous PSD have not separated performance based on yes-no decision choices.

### Permanent Memory – Word Association Task

The Word Association task examined processes involved in the search of permanent, semantic memory for words. In the normal sleep condition, the usual main effect of the strength of word association was observed, accuracy of responding being higher and speed of responding faster following target words that were strongly associated to previously presented prime words. The strength of association effect was also observed in both the total and PSD conditions. Performance differences as a result of the strength of word association are therefore very robust and seemingly immune even to the effects of 24 h of TSD. TSD did affect overall results, independent of the strength of association. Accuracy significantly decreased and failures to respond significantly increased following TSD regardless of the strength of word association between the target and the prime. The reduction in accuracy is similar to that reported by López Zunini et al. (2014) except that in their case, the difference compared to the normal sleep condition was not significant. In the present PSD study, sleep loss was not associated with an overall significant decline in accuracy of target detection, these results replicating the Tavakoli et al. (2015) findings. Sleep loss whether total or partial did not have a significant effect on RT to the target words.

Sleep deprivation may thus have only a small effect on the Word Association task and this effect is independent of the strength of word association. The well-documented priming effect thus appears to be preserved regardless of the amount of sleep loss. This may be because semantic activation, spreading from the prime to the target, is carried out automatically in nature (Collins and Loftus, 1975). Still, the present study was designed to allow the use of an effortful predictive, expectancy-based strategy (Posner and Snyder, 1975; Neely, 1976; Becker, 1980) employing a sufficiently long period of time between the presentation of the prime and subsequent target to permit prediction of the target word. Further, the task required subjects to intentionally make a word association decision. There is still much debate about the extent of use of automatic and controlled processing in priming tasks. de Wit and Kinoshita (2015), for example, question whether a common strategy is used in all priming-word association tasks and argue that the specific processes will vary depending on the nature of the task. It is also possible that subjects who are not sleep-deprived (i.e., normal sleep) could use a controlled, effortful strategy whereas those who are sleep-deprived are forced to rely on a more automatic, effortless strategy in order to maintain a relatively high level of performance.

### Conclusion

Taken together, our results reveal that TSD and PSD have various detrimental effects on performance measures for all tasks tested in this study. In agreement with previous accounts in the sleep deprivation literature, TSD was associated with a significant decline in performance, usually in accuracy and at times, in RT, for all tasks. Our expectation that the overall adverse effects on performance would be reduced for PSD, in comparison to those of TSD, also seemed to be corroborated. PSD led to a general cognitive decline, but did not reach statistical significance in some of the performance measures of particular tasks.

There are also limitations to the present PSD study. The sample size, although relatively large, may still not have provided adequate statistical power. On the other hand, if a much larger sample size is required to obtain significance, the differences may be too small to be of practical value in the applied and clinical setting. As the TSD study clearly demonstrated, the complete absence of even one night of sleep can have a strong effect, while 4 h of sleep may obviate at least its larger effects. The optimal amount of sleep remains a contentious issue in sleep research.

A sleep-delay procedure was used in the present study, subjects delaying the onset of sleep by 4 h later than normal. The alternative early waking procedure, subjects waking 4 h earlier than normal, has also been employed in other studies. The effects of delayed sleep onset compared to early awakening may not be identical (Zerouali et al., 2010). This is not incidental. Delaying sleep onset (“staying up late”) is a very common cause of sleep loss in almost all young adults. Among older adults, the opposite, an inability to remain asleep, is often reported. Also, this study was designed to determine the effects of partial sleep loss over a single night, again a common occurrence among those tested in applied and clinical settings. Deterioration in performance may, however, accumulate over 4–5 nights of PSD (Dinges et al., 1997; Van Dongen et al., 2003; Drummond et al., 2012). Such chronic sleep loss is often observed among sleep disorders (e.g., insomnia, restless leg syndrome, obstructive sleep apnea syndrome), neurological and psychiatric conditions (e.g., major depressive disorder, Parkinson’s disease) and occupational demands such as shift work (Colton and Altevogt, 2006; Åkerstedt and Wright, 2009). Our findings do, nevertheless, generally support the notion that acute TSD and PSD, even if it is few as 4 h, may affect any cognitive task that requires sustained attention and vigilance for successful performance, but also interact with specific cognitive processes. Because sleepiness and fatigue appear to have a direct effect on performance, subjects participating in cognitive studies should, at the very least, be screened for sleep difficulties and sleep-related habits.

## Ethics Statement

This study was carried out in accordance with the Canadian Tri-Council guidelines (Health, Natural, and Social Sciences) for ethical conduct involving human participation. These guidelines are similar to those of the Declaration of Helsinki. All subjects gave written informed consent. The protocol was approved by the University of Ottawa’s Health Sciences and Science Research Ethics Board.

## Author Contributions

The three authors (DG, JDK, and KC) confirmed that they each made substantial contributions to the conception or design of the work and the acquisition, analysis, and interpretation of data for the work; drafted the work and revised it critically for important intellectual content; approved the final version to be published; and agreed to be accountable for all aspects of the work in ensuring that questions related to the accuracy or integrity of any part of the work are appropriately investigated and resolved.

## Conflict of Interest Statement

The authors declare that the research was conducted in the absence of any commercial or financial relationships that could be construed as a potential conflict of interest.

## Footnotes

1 Given the nature of this task, some authors would prefer to describe it as a test of short-term rather than working memory. However, because most studies of sleep deprivation employ “working” memory as a descriptor, this is the convention that will be used in the present study.
